# How Does Host Carbon Concentration Modulate the Lifestyle of Postharvest Pathogens during Colonization?

**DOI:** 10.3389/fpls.2016.01306

**Published:** 2016-09-01

**Authors:** Dov B. Prusky, Fangcheng Bi, Juan Moral, Shiri Barad

**Affiliations:** ^1^Department of Postharvest Science of Fresh Produce, Agricultural Research Organization, The Volcani CenterBeit Dagan, Israel; ^2^Institute of Fruit Tree Research, Guangdong Academy of Agricultural Sciences, Key Laboratory of South Subtropical Fruit Biology and Genetic Resource Utilization, Ministry of AgricultureGuangzhou, China; ^3^Departamento de Agronomía, Universidad de CórdobaCórdoba, Spain

**Keywords:** small effector molecules, pH regulation, pathogenicity, postharvest susceptibility, colletotrichum, penicillium

## Abstract

Postharvest pathogens can penetrate fruit by breaching the cuticle or directly through wounds, and they show disease symptoms only long after infection. During ripening and senescence, the fruit undergo physiological processes accompanied by a decline in antifungal compounds, which allows the pathogen to activate a mechanism of secretion of small effector molecules that modulate host environmental pH. These result in the activation of genes under their optimal pH conditions, enabling the fungus to use a specific group of pathogenicity factors at each particular pH. New research suggests that carbon availability in the environment is a key factor triggering the production and secretion of small pH-modulating molecules: ammonia and organic acids. Ammonia is secreted under limited carbon and gluconic acid under excess carbon. This mini review describes our most recent knowledge of the mechanism of activation of pH-secreted molecules and their contribution to colonization by postharvest pathogens to facilitate the transition from quiescence to necrotrophic lifestyle.

## Introduction

The resistance of unripe fruit to pathogen infection and colonization after harvest is considered a dynamic process that is modulated during host maturation and ripening. In many postharvest pathogens, disease symptoms occur long after the initial stages of infection when the pathogen is quiescent. During ripening of the host, the quiescent biotrophic infection resulting from fruit penetration directly or through wounds becomes active and develops into necrotrophic colonization that manipulates the host’s physiological response (Denison et al., 1995; Calvo et al., 2002; Caracuel et al., 2003b; O’Meara et al., 2010). For successful colonization, a pathogen must be able to overcome the host’s defenses and initiate attack under prevailing physiological and environmental conditions. During this period, the pathogen must trigger pathogenicity factors that macerate host tissues and release the nutrients required to sustain its development. Since both the host and the pathogen are living entities, the conditions imposed by the host are critical to inducing susceptibility and activating the pathogen quiescent stage. While the mechanism of pH modulation by fungal metabolism has been thoroughly reported, no specific studies have indicate the effect of host pH on fungal pathogenicity. Furthermore fruit ripening and host susceptibility is accompanied by significant sugar accumulation, pH change and many other host changes that affect fungal pathogenicity and have not been independently studied (Prusky, 1996). In this mini review, we analyze the conditions that modulate the pathogen’s initial stages of colonization by pH modulation of the host.

## Postharvest Pathogens and pH Modulation

The ability of postharvest pathogens to alter pH locally was initially described for *Colletotrichum gloeosporioides*, and then extended to some other pathogens, such as *Alternaria alternata, Botrytis cinerea, Penicillium expansum, Penicillium digitatum, Penicillium italicum, Phomopsis mangiferae, Monilinia fructicola*, and *Fusarium oxysporum* (Prusky et al., 2001, 2004; Rollins and Dickman, 2001; Eshel et al., 2002a,b; Manteau et al., 2003; Davidzon et al., 2010; Miyara et al., 2010, 2012).

Ambient alkalization by fungi is achieved by their active secretion of ammonia, which results from the activation of proteases followed by deamination of amino acids (Jennings, 1989; Miyara et al., 2010). Ammonium accumulation has been detected in association with pathogenicity of many *Colletotrichum* species, including *C. gloeosporioides, C. acutatum, C. higginsianum, C. graminicola*, and *C. coccodes* (Alkan et al., 2008; Dieguez-Uribeondo et al., 2008; Miyara et al., 2010; O’Connell et al., 2012), *A. alternata* (Eshel et al., 2002a,b), and *F. oxysporum* (Miyara et al., 2012). The ammonium secreted by these species alkalizes the host tissue, and its concentration can reach approximately 5 mM, as found in decayed avocado, tomato, and persimmon fruit (Eshel et al., 2002a,b; Alkan et al., 2008; Miyara et al., 2010). In each case with *Colletotrichum* spp., increased ammonium accumulation has been related to enhanced pathogenicity (Alkan et al., 2008, 2009; Miyara et al., 2010). In the case of *A. alternata*, ammonium accumulation led to a 2.4 pH unit increase in several hosts—tomato, pepper, melon, and cherry (Eshel et al., 2002a,b). Interestingly, ammonia accumulation and pH increase were not correlated across host species, suggesting that pH increase in each host depends on a complex interaction that involves the buffer capacity of the tissue, nitrogen, and carbon availability, and the fruit’s initial pH (Eshel et al., 2002b). Indeed, fruit differ in their buffer capacity and pH. However, low pH has been found to activate higher ammonia production and secretion in *Colletotrichum* spp. (Kramer-Haimovich et al., 2006; Alkan et al., 2008).

In contrast, other pathogenic fungi, such as *P. expansum, P. digitatum, P. italicum* (Prusky et al., 2004), *Phomopsis mangiferae* (Davidzon et al., 2010), *Aspergillus niger* (Ruijter et al., 1999), *B. cinerea* (Manteau et al., 2003), and *Sclerotinia sclerotiorum* (Bateman and Beer, 1965) use tissue acidification in their attack. Tissue acidification is enhanced by the secretion of organic acids and/or H^++^ excretion. *S. sclerotiorum* and *B. cinerea* decrease host pH by secreting significant amounts of oxalic acid (OA; Rollins and Dickman, 2001; Manteau et al., 2003); gluconic acid (GLA) is secreted by *Phomopsis mangiferae* (Davidzon et al., 2010), and combinations of gluconic and citric acids are mainly secreted by *Penicillium* (Prusky et al., 2004) and *Aspergillus* (Ruijter et al., 1999). In *P. expansum*, reduced GLA accumulation has been related to reduced pathogenicity (Barad et al., 2014).

In both cases, alkalization or acidification of the environment by the secretion of ammonia by *Colletotrichum* or organic acid by *Penicillium*, respectively, clearly modulates (activating or repressing) pathogenicity factors. *P. expansum* acidifies the host tissue to pH levels of 3.5–4.0, at which polygalacturonase (*pg1*) transcription is significantly enhanced (Prusky et al., 2004). Similarly, in *C. gloeosporioides, pelB* (encoding pectate lyase) is expressed and secreted *in vitro* at pH levels higher than 5.7, similar to the pH values present in decaying tissue (Prusky et al., 1989; Yakoby et al., 2000, 2001). Analysis of endoglucanase 1 gene expression in *A. alternata* showed maximal expression at pH levels higher than 6.0, i.e., values similar to those found in the decayed tissue in which maximal virulence was observed (Eshel et al., 2002b). This suggests that postharvest pathogens modulate the expression of genes contributing to pathogenicity according to environmental pH-inducing conditions.

## Gene Modulation of Fungal Pathogenicity Factors

What is the mechanism governing fungal modulation of pH-responsive genes? PacC is a transcription factor that regulates gene expression under increasing alkaline conditions. Previous work in the model fungal system *Aspergillus* has suggested that PacC responds to external pH to enable fungal survival under varied pH conditions (Penalva et al., 2008; Selvig and Alspaugh, 2011). Moreover, in fruit fungal pathogens, *pacC* knockout significantly reduces pathogenicity (Miyara et al., 2008; Zhang et al., 2013), suggesting that this transcription factor not only modulates genes for fungal survival, but contributes to pathogenicity as well. The reports that the pathogen may modulate pH by increasing or decreasing the pH of the environment, as described in Section “Postharvest Pathogens and pH Modulation,” suggest that PacC shows dual regulation of pathogenicity genes (activation and repression) under pH change. Thus, it is likely that fungi with different pH preferences contain an arsenal of both alkaline– and acid-regulated genes to exploit changing pH conditions. Alkan et al. (2013) characterized alkaline– and acid-expressed genes. Those modulated genes encoded transporters, antioxidants and cell wall-degrading enzymes (CWDEs) (Alkan et al., 2013). Transporters, including those involved in sulfate, potassium, carboxylic acid, and ammonium transport, are likely to be controlled by pH due to the direct pH effect on the charge of inorganic or organic acid ions. The upregulation of transporters may compensate for changes in ionic differences between intracellular and extracellular regions to restore fungal homeostasis under changing pH (Bensen et al., 2004). The pH shifts also seem to affect cellular redox status, as exemplified by changes in antioxidants that include catalase activity and hydrogen peroxide catabolic process. Major components of PacC regulation in *C. gloeosporioides* are CWDE pathogenicity factors. Genes that are shown here to be affected by PacC include *pelB*, and those encoding cellulase, α-mannosidase and 1,4-β-xylanase activity. These findings extend the repertoire of pH-modulated CWDEs from the previously identified PelB in *C. gloeosporioides*, endoglucanases in *Alternaria alternata* (Yakoby et al., 2000; Prusky et al., 2001, 2004; Eshel et al., 2002a), and polygalacturonases *Bcpg*1-6 in *B. cinerea* (Wubben et al., 2000; ten Have et al., 2001).

What is interesting to note is that gene families with members of similar functionality were both up- and downregulated by PacC (Alkan et al., 2013). This indicated that similar functions might occur under alkaline and acidic conditions, including CWDE activity. The differential pH regulation of genes with similar activities suggests that they are selectively activated on the basis of their optimal enzymatic pH activity, allowing the fungus to cope with variable pH conditions and make optimal use of the available enzymes.

While PacC has been reported as a gene regulator under alkaline conditions, a recent publication by Barad et al. (2016) showed that the *pacC* transcript can be activated under acidic conditions in *P. expansum*. Electrophoretic mobility shift assay (EMSA) of *P. expansum* PacC, together with antibodies against the different cleaved proteins, showed that PePacC is not protected against proteolytic signaling at pH 4.5 compared to pH 7.0. Moreover, Barad et al. (2016) observed that ammonia is not produced only by alkalizing pathogens, but by acidifying pathogens as well, under specific growth conditions, at reduced carbon levels and at the leading edge of the colonized area (Barad et al., 2016). Ammonia did not further enhance PacC proteolytic cleavage but did enhance activation of *palF* transcript in the PaL pathway under acidic conditions. The PaL pathway represents a key process regulating PacC cleavage (Diez et al., 2002). Ammonia accumulation in the host environment by the pathogen under acid pH may be a regulatory cue for *pacC* activation, toward accumulation of pathogenicity factors. This process has not been investigated in other acidifying pathogens. However, similar processes may be occurring there as well.

The results obtained under acidification and alkalization conditions are consistent with the observation that ΔpacC mutants of *C. gloeosporioides, C. acutatum, F. oxysporum, P. expansum*, and *S. sclerotiorum* are less virulent than the wild type (Caracuel et al., 2003a; Rollins, 2003; You et al., 2007; Miyara et al., 2008; Zhang et al., 2013; Barad et al., 2014). This suggests the importance of gene regulation by PacC in acidifying and alkalizing pathogens. It indicates that PacC controls enzyme fine-tuning so that the optimum repertoire will be expressed at any given pH. That is probably how transporters and antioxidants maintain homeostasis and expression of pathogenicity factors for orchestration of the genomic arsenal under changing pH. Hence, at each pH, the fungus is likely to express an optimal gene combination. Those acid-expressed genes are crucial for *P. expansum* and *B. cinerea* pathogenicity because the pathogenicity thrives at low pH. Reciprocally, in fungi that alkalinize the environment, such as *C. gloeosporioides* and *A. alternata*, PacC will be activated only after the fungi raise the surrounding pH. Because fungi are likely to encounter a broad spectrum of initial environmental pH, broad conservation of pH responses may be activated to justify a preferred pH for pathogenicity.

## Modulating the Activation of Small Secreted Molecules

The pathogens’ ability to secrete pH-regulating molecules, on the one hand, and the transcriptome analysis of PacC-modulated genes, on the other, has revealed that pH may regulate the arsenal of pathogenicity factors. However, previous reports in most postharvest pathogens have shown that a given pathogen has a single, specific lifestyle by which it modulates its host pH, and the same pathogen was usually not found to be able to act in the opposite direction (**Table 1**). The questions are: how specific are the pH-regulating patterns for each particular fungal species during pathogenicity, and what is the signal that may differentially activate the specific pH modulation during colonization?

**Table 1:** Fungal pathogens and small secreted molecules that modulate pH for the activation of pathogenicity factors.

Pathogens	Alkalizers	Acidifiers
*Colletotrichum*	Ammonia	
*Alternaria*	Ammonia	
*Fusarium*	Ammonia	
*Penicillium*		Gluconic acid
*Phomopsis*		Gluconic acid
*Monilinia*		Gluconic acid
*Sclerotinia*		Oxalic acid
*Botrytis*		Oxalic acid

One of the significant changes observed in fruit during ripening is an increase in sugar content. Sugars are one of the major constituents responsible for tomato fruit quality, accounting for some 50% of the dry matter (Hulme, 1971; Prusky, 1996). In tomato the total sugar content increases progressively during ripening from the mature-green to red-ripe stage. The sucrose content of bananas also changes from a high concentration of starch to a higher concentration of sucrose during ripening (Hulme, 1971; Prusky, 1996).

**FIGURE 1:**
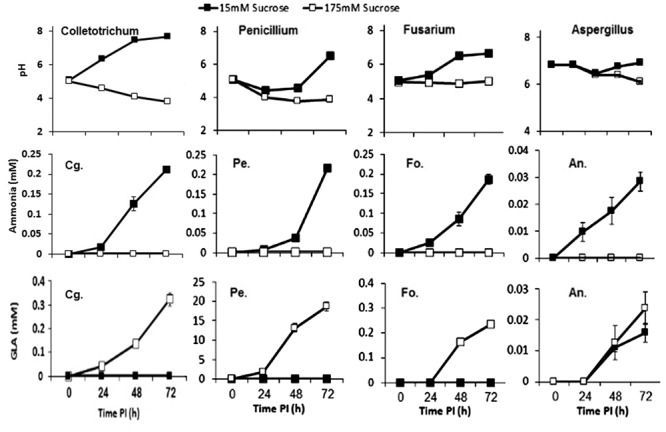
**Effects of carbon level on the induction of alkalization or acidification of medium by *Penicillium expansum* (Pe.), *Fusarium oxysporum* (Fo.), *Aspergillus nidulans* (An.), and *Colletotrichum gloeosporioides* (Cg.).** Fungal mycelia were grown in primary rich medium for 2–3 days and then transferred to secondary medium containing sucrose at 15 mM (▪) or 175 mM (□), adjusted to pH 5, for 72 h (Bi et al., 2016).

In a recent work by Bi et al. (2016), it was reported that postharvest pathogens such as *C. gloeosporioides, P. expansum, Aspergillus nidulans* and *F. oxysporum* can cause either alkalization or acidification of their environment. The acidification was induced by all pathogens under carbon excess, e.g., 175 mM sucrose; in contrast, alkalization occurred under conditions of carbon deprivation, e.g., less than 15 mM sucrose. The carbon source was metabolized by glucose oxidase (GOX2) to GLA, contributing to medium acidification, while catalyzed deamination of non-preferred carbon sources, such as the amino acid glutamate, by glutamate dehydrogenase 2 (GDH2) resulted in the secretion of ammonia. Interestingly, this type of response was similar in *C. gloeosporioides, P. expansum, A. nidulans*, and *F. oxysporum*, suggesting that carbon response is concentration-dependent rather than pathogen-dependent (Bi et al., 2016) (**Figure 1**).

**FIGURE 2:**
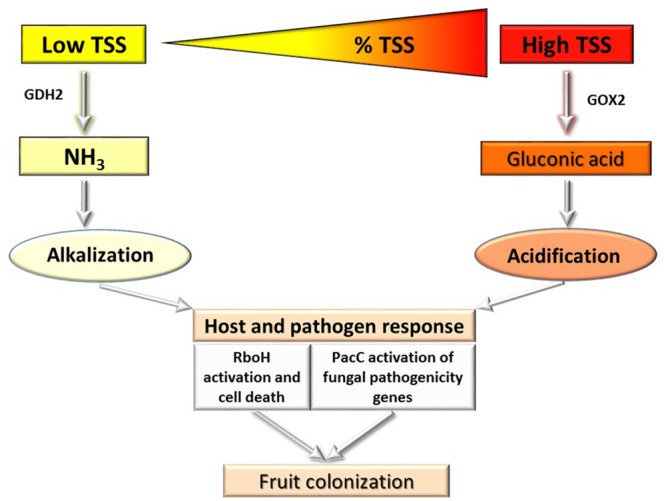
**Diagram of the effect of carbon levels in the fruit on the induction of alkalization and acidification, modulation of PacC and colonization by postharvest pathogens**.

Can different host nutritional conditions, such as increasing sugar levels during fruit ripening, modulate the type of small effector molecules secreted by fungi to modulate host pH? Fungi possess sensitive gene-regulatory mechanisms to respond to nutrient fluctuations in the environment, as occur in ripening fruit or growing plants. Nutritional availability at the initial stages of germination and growth is certainly different from that during necrotrophic colonization, where nutrients are available in excess (Bi et al., 2016). Lack of nutrient availability at the leading edge of the colonized tissue of ripening fruit induces ammonia accumulation by *C. gloeosporioides* (Miyara et al., 2010). With low sugar concentrations, the importance of glutaminolysis for cell energy supply is clear, and ammonia is generated as a byproduct of the glutaminase and glutamate dehydrogenase synthesis reactions (Newland et al., 1990). Similarly, exposure of *P. expansum* spores to natural acidic conditions on the wounded fruit peel enhances its germination and biomass development (Barad et al., 2012). Under high glucose/sucrose concentrations in ripe fruit, sugar may be oxidized to CO_2_ via tricarboxylic acid, with high rates of glycolysis and the production of organic acids that contribute to the secretion of metabolites that decrease host pH (**Figure 2**). Bi et al. (2016) found accumulation of ammonia by *C. gloeosporioides* and enhanced alkalization during pathogenicity on tomato, whose total sugar content reached 6%. However, in plum fruit, with a sugar concentration of at least 14%, the same pathogen did not accumulate ammonia. On the contrary, in plum, accumulation of GLA by *C. gloeosporioides* was twice as high as in inoculated tomato, suggesting that during host colonization, the balance between ammonia and GLA accumulation by the same pathogen also determines the final pH of the host environment.

Understanding the genetic pathways that regulate the responses of pathogenic fungi to their environment is paramount to developing effective disease–prevention strategies. Pathogens use specific gene-induction pathways to metabolize a wide range of carbon and nitrogen compounds, but this colonization is moderated by two global regulatory systems that ensure the preferential utilization of a few favored carbon and nitrogen sources. Carbon catabolite repression (CCR) is a global regulatory mechanism found in a wide range of microbial organisms; it ensures the utilization of preferred carbon sources, such as glucose, over less favorable ones. However, little is known about the components of CCR that interact with pH-modulating nitrogen systems: CCR operates via the negatively acting zinc finger repressor CreA to ensure that glucose is utilized preferentially, by preventing the expression of genes required for the metabolism of less preferred carbon sources (Fernandez et al., 2012, 2014). According to Bi et al. (2016), CreA is induced at high sucrose concentrations where GLA accumulation is induced and ammonia production is repressed. How is this system activated? This question is of high importance for understanding the differential pH response and the consequent expression of genes that modulate pathogenicity under dynamic pH and colonization conditions (**Figure 2**).

## Author Contributions

DP wrote the manuscript and FB contribute to test the effect carbon on *Colletotrichum* and SB contribute to test the effect of carbon on *Penicillium.* JM reviewed and discussed the final revised version.

## Conflict of Interest Statement

The authors declare that the research was conducted in the absence of any commercial or financial relationships that could be construed as a potential conflict of interest.
